# The Efficacy of Cognitive Behavioral Therapy for Tic Disorder: A Meta-Analysis and a Literature Review

**DOI:** 10.3389/fpsyg.2022.851250

**Published:** 2022-03-24

**Authors:** Songting Shou, Yuanliang Li, Guohui Fan, Qiang Zhang, Yurou Yan, Tiying Lv, Junhong Wang

**Affiliations:** ^1^Department of Pediatrics, Dongzhimen Hospital, Beijing, China; ^2^Graduate School, Beijing University of Chinese Medicine, Beijing, China; ^3^Department of Integrative Oncology, China-Japan Friendship Hospital, Beijing, China; ^4^Institute of Clinical Medical Sciences, China-Japan Friendship Hospital, Beijing, China

**Keywords:** cognitive behavioral therapy, habit reversal therapy, comprehensive behavioral intervention for tics, tic disorders, meta-analysis

## Abstract

**Background:**

At present, tic disorder has attracted the attention of medical researchers in many countries. More clinicians choose non-drug therapy, especially cognitive-behavioral therapy (CBT) because of the cognitive side effects of drug therapy. However, few studies had assessed its efficacy. It is necessary to have a more comprehensive understanding of the literature quality of CBT and its intervention effect.

**Methods:**

In this study, MEDLINE, Embase, and Cochrane were searched from the beginning to June 15, 2021 to study the efficacy of -CBT on tic disorder. Only studies using the Yale Global Tic Severity Scale (YGTSS) and the control group were included.

**Results:**

A total of 12 randomized controlled trials (RCTs), including 536 patients with tic disorders, were identified. The results showed that the effect of CBT was better than that of the control group. The pooled standardized mean difference (SMD) was −0.34 (95% CI: −0.61, −0.07). The effect size of CBT differs from different intervention conditions. In seven studies, the subjects’ motor tic scores were counted. The sample size of the experimental group was 224 and that of the control group was 218. The pooled SMD was −0.43 (95% CI: −0.75, −0.11). Seven studies counted the vocal tic scores of subjects, 224 in the experimental group and 218 in the control group. The pooled SMD was −0.22 (95% CI: −0.54, −0.11). Seven studies counted the tic impairment scores of subjects, 220 in the experimental group and 214 in the control group. The pooled SMD was −0.48 (95% CI: −0.73, −0.23).

**Conclusion:**

The literature shows that different CBTs can significantly reduce the total score of tic disorder and the score of motor tic, but cannot significantly reduce the score of vocal tic. In the future, more new interventions were needed to improve the symptoms of different patients, especially vocal tic.

## Introduction

Tic disorder is defined as a neurodevelopmental disorder that occurs or motor extraction lasts for more than 1 year (American Psychiatric Association [APA], 2013). Studies have pointed out that tic disorder is a syndrome related to psychology, involving emotional changes, psychological problems, and behavioral problems (O’Hare et al., 2016). One of the behavioral problems is that they often speak inappropriate language on inappropriate occasions, which may lead to their loss of work and affect their life and labor ability (Davis et al., 2004). Relevant studies have shown that from the perspective of patients, there is no difference between the issuance of tic disorder movements and the issuance of normal movements in their life (Ganos et al., 2021). Other studies attribute tic disorder, nail-biting, hair-pulling addiction, and other diseases to personal habits. Therefore, patients can be treated through the cognitive-behavioral therapy (CBT), such as habit reversal training (HRT) (Bate et al., 2011), the comprehensive behavioral intervention of tic (CBIT) (Zimmerman-Brenner et al., 2021), neurofeedback (Sukhodolsky et al., 2020), and so on. HRT includes five exercises (Azrin and Nunn, 1973). The first exercise is called awareness training, which trains patients to perceive their feelings, environment, frequency, and high-risk situations when they tic (Ganos et al., 2021). Relaxation training is to teach patients to resist the stress they encounter (Ganos et al., 2021). Competition response training is to train patients to form a new habit, so as to prevent the emergence of tic. This new habit needs to be not easy to be found by others in life and can maintain a certain duration (Ganos et al., 2021). Motivation training is achieved by stimulating the patient’s own inner motivation. The main method is to let them understand the harm of convulsions, encourage others, and let patients show others control of convulsions (Teng et al., 2006; Bate et al., 2011). Generalization training is to train patients to imagine and rehearse in the brain to control convulsions when they are about to appear (O’Donohue et al., 2003). Later, researchers gradually developed richer treatment schemes in practice, such as CBIT. Based on HRT, other researchers have also developed different treatment schemes, such as coexistence with convulsions (living with tic; LWT) (McGuire et al., 2015) and anger control training (ACT) (Sukhodolsky et al., 2009), and added training modules for patients’ families in the cognitive-behavioral treatment scheme.

Some drugs are used to treat tic disorder, but these drugs have certain side effects. In drug treatment, clinicians often choose risperidone, clonidine, and aripiprazole for treatment, but the specific drug is the preferred drug, and the survey results of different studies are inconsistent (Chadehumbe et al., 2011; McNaught and Mink, 2011). Clonidine is A-2 agonist, and its main side effect is sedation (Goetz, 1992; Weisman et al., 2013). It is thought to reduce tics through the resultant decrease of norepinephrine release and turnover (Song et al., 2017). Risperidone is an antipsychotic drug, and its side effect is sedation (Gaffney et al., 2002). It has a high affinity for the dopamine D2 receptor and 5-HT2 receptor. It may reduce tic by regulating dopamine and 5-HT (Roessner et al., 2011). The side effects of haloperidol are sedation and cognitive retardation (Curtis et al., 2009). The researchers believe that it reduces convulsions by blocking the D2 dopamine receptor in the striatum (Bressan et al., 2004). The cannabinoid is another drug that is used to treat tic disorder. It is currently believed that it also reduces tic by regulating dopamine. A study of 20 people taking cannabinoids for a long time showed that 19 people had side effects, such as cognitive function (Romero et al., 2015). Because the pathogenesis and mechanism of tic disorder are not clear, it is difficult to determine the cause of the efficacy of these drugs. Since the side effects of these drugs are mainly sedation and affect cognitive function, it can be speculated that these drugs may reduce symptoms by suppressing some normal brain function. Therefore, it is possible that too strong cognitive function is the cause of tic disorder, which may lead to patients being too sensitive to the changes in the surrounding environment, resulting in various psychological changes, resulting in physical reactions and behavioral problems. This situation is the goal of CBT treatment. Instead of using drugs to inhibit the cognitive function of patients, it is better to correctly guide the cognitive function of patients through CBT. Therefore, this study only includes CBT research and summarizes the role of CBT in the treatment of tardive dyskinesia (TD).

At present, some meta-analysis studies (McGuire et al., 2014; Yu et al., 2020) have determined the therapeutic effect of some CBT, but some problems need to be further studied. First, some new findings can be added to the meta-analysis to help clinicians make more rigorous decisions (McGuire et al., 2014; Chen et al., 2020). Secondly, the efficacy of different CBTs on different tic symptoms needs to be compared (O’Donohue et al., 2003). Therefore, in this study, we conducted a meta-analysis to determine the efficacy of CBT in patients with a tic disorder. We also compared the efficacy of CBT in different aspects of tic disorder. The control group of the study involved included group-Educational Intervention for Tics (EIT), sham-control, psychoeducation (PE), psychoeducation and supportive therapy (PST), supportive psychotherapy (SP), treatment-as-usual (TAU), and waitlist. The main content of the intervention methods of EIT, PE, PST, SP, and other psychological education groups is to teach patients how to face psychological problems, such as stress and anxiety (Zimmerman-Brenner et al., 2021). The sham control group is mainly to isolate the electroencephalographic (EEG) biofeedback communication between the patient and the display, so as to realize the comparison with the experimental group. The experimental group can complete the relevant tasks through the EEG signal control software, while the EEG signal control software in the sham control group cannot complete the relevant tasks (Sukhodolsky et al., 2020). TAU group includes parental education, clinical monitoring of specific symptoms, drug management, school counseling, etc. (Sukhodolsky et al., 2009). These methods can alleviate the psychological problems of TD patients to some extent, but the symptom relief may not be obvious.

## Methods

### Search Strategy and Selection Criteria

MEDLINE, Embase, and Cochrane were searched from the beginning to June 15, 2021. Search terms included “cognitive behavior therapy” and “tic disorder” along with numerous other related terms. We limited the study language to English. The full search strategies are detailed in the Supplementary Material. We also reviewed the reference lists of eligible studies and previous evidence summaries to identify additional literature.

Two reviewers (SS and YL) working independently considered the potential eligibility of each of the abstracts generated by the search strategy. Full-text articles were obtained unless both reviewers decided that an abstract was ineligible. Each full-text report was assessed independently for final study inclusion. Disagreements about the inclusion of full-text articles were resolved by consensus.

#### Inclusion and Exclusion Criteria

Our study only included randomized controlled trials (RCTs), and the intervention form of the experimental group must be CBT. The intervention forms of the comparison group included routine nursing, routine medical education, relaxation training, psychological education, supportive therapy, waiting list, or placebo treatment. The outcome was evaluated within 1 month after treatment. The evaluation tool was the Yale Global Tic Severity Scale (YGTSS). Studies were excluded in which the control group also used cognitive behavior treatment studies or did not give any score in the YGTSS.

### Data Extraction

We extracted the following data from various studies: (1) the first author; (2) year of publication; (3) sample size; (4) mean and standard deviation (SD) of age; (5) outcome indicators; (6) intervention methods; and (7) duration of intervention. Draw the above information into a standard form, which will be evaluated by two reviewers, and form a unified opinion after negotiating different opinions. Researchers check each other to ensure the accuracy of test data entry.

### Effect Size

Review Manager 5.3 software was used to analyze various data. The efficacy was evaluated according to the mean and the standard mean difference (Weisman et al., 2013). The differences between the two groups were compared by comparing the data of the experimental group and the control group. The total tic score, motor tic score, sound tic score, and tic impairment score of the two groups were drawn by forest plots.

### Quality Assessments

The quality of literature was evaluated according to the quality evaluation criteria specified in the Cochrane bias risk assessment tool (Higgins et al., 2008). The evaluation was conducted from selection (such as random sequence and allocation concealment), implementation (such as blinding by researchers and subjects), measurement (blind evaluation of study outcomes), follow-up (the integrity of outcome data), reporting (selective reporting of study results), and other (other sources of bias). The judgment results of “low-risk bias,” “high-risk bias,” and “unclear” were made according to the bias risk assessment criteria. It is displayed in different colors (green, red, and yellow).

### Statistical Analyses

Revman5.3 software was used for meta-analysis to quantitatively synthesize the data. The standardized mean difference (SMD) was used as the effect scale index for the measurement data, which was expressed by 95% confidence intervals (CI). According to the Z or U value or chi-square value, the probability *p*-value under this statistic is obtained, and the significance level is set to 0.05. If *p* < 0.05, the combined effect of multiple studies is statistically significant. If there is no statistical heterogeneity between studies (*I*^2^ ≤ 50% or *p* ≥ 0.10), the fixed-effect model is adopted. If there is heterogeneity (*I*^2^ > 50% or *p* < 0.10), subgroup analysis and sensitivity analysis are carried out according to the factors that may cause heterogeneity, but the heterogeneity cannot be eliminated, the random effect model is adopted. Publication bias analysis was performed by stata mp 14.

## Results

### Included Studies

**FIGURE 1:**
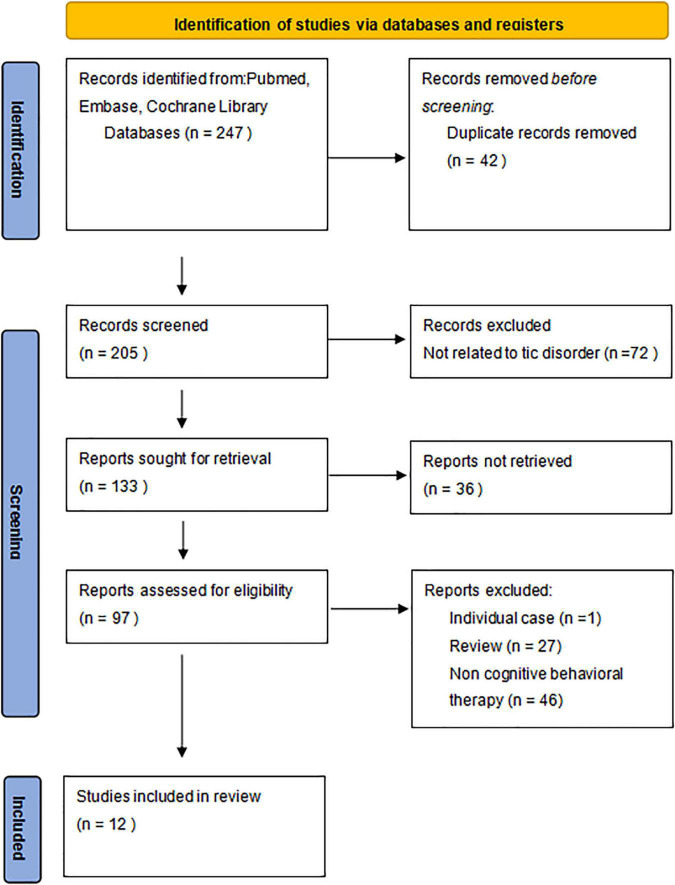
The Preferred Reporting Items for Systematic Reviews and Meta-Analyses (PRISMA) flowchart of study identification, screening, assessment of eligibility, and inclusion for synthesis.

Two hundred and forty seven articles were identified by the search strategy, of which 12 met the criteria for full-text review (Figure 1). The characteristics of the included studies are summarized in the Supplementary Material. In total, 12 RCTs that include 565 patients were met the eligibility criteria. Among the 12 RCTs, 4 studies by Wilhelm et al. (2003), Deckersbach et al. (2006), Yates et al. (2016), and Rizzo et al. (2018) involved HRT. Five studies by Piacentini et al. (2010), Wilhelm et al. (2012), Ricketts et al. (2016), and Zimmerman-Brenner et al. (2021) involved CBIT. Three studies involved some other cognitive-behavioral treatments, consisting of ACT (Sukhodolsky et al., 2009), LWT (McGuire et al., 2015), and neurofeedback (Sukhodolsky et al., 2020). The author, sample size, intervention methods, and related characteristics are listed in Table 1.

**TABLE 1:** Basic characteristics of the included studies.

References	Nationality	Sample size of intervention group	Sample size of control group	Intervention methods	JADAD
Zimmerman-Brenner et al., 2021	Israel	23	23	CBIT/EIT	7
Sukhodolsky et al., 2020	America	23	23	Real-NF/Sham control	7
Chen et al., 2020	China	12	12	CBIT/PE	7
Rizzo et al., 2018	Rome	25	24	hrt/PST	5
Ricketts et al., 2016	United States	12	8	CBIT/Waitlist	5
Rizzo et al., 2018	Rome	25	24	hrt/PST	5
Yates et al., 2016	United Kingdom	17	16	HRT/SP	4
McGuire et al., 2015	United States	12	12	LWT/Waitlist	4
Wilhelm et al., 2012	United States	63	59	CBIT/PST	6
Piacentini et al., 2010	United States	61	65	CBIT/PST	4
Sukhodolsky et al., 2009	United States	13	13	ACT/TAU	5
Wilhelm et al., 2003	United States	16	7	HRT/PST	4

*CBIT, comprehensive behavioral intervention for tics; HRT, habit reversal therapy; LWT, living with tic; SP, supportive psychotherapy; PE, psychoeducation; NF, neurofeedback; PST, psychoeducation and supportive therapy; ACT, anger control training; TAU, treatment-as-usual; EIT, educational intervention for tics.*

### Risk of Bias

**FIGURE 2:**
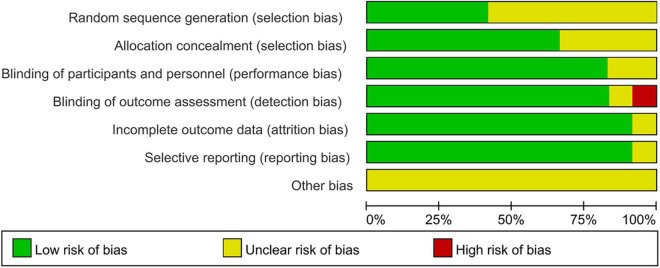
Risk of bias in the included trials.

**FIGURE 3:**
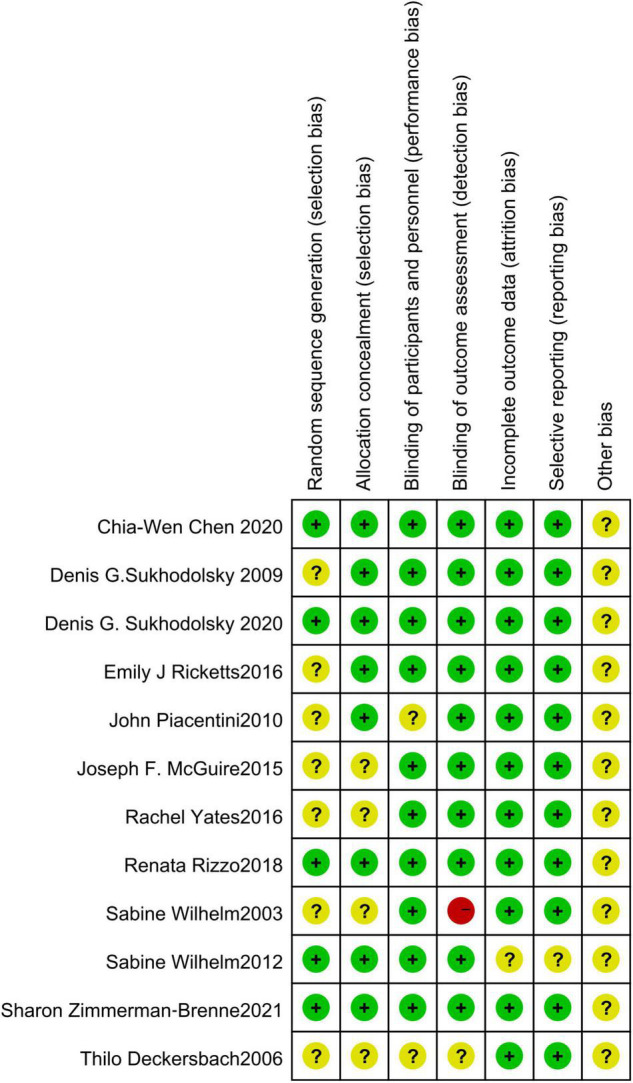
Risk of bias in individual studies. +, low risk of bias; ?, unclear risk of bias; –, high risk of bias.

Of the 12 RCTs included in this study, 7 did not describe how to generate random sequences, 4 did not describe the allocation and concealment, 2 did not describe how to use the blind method for participants and personnel, 1 did not describe how to use the blind method to evaluate the outcome data, the outcome data of 1 study assessed that blind method was of high risk, and 1 study may have incomplete outcome data, one study may have selective reporting bias. There may be some bias in these studies, but it is not possible to assess whether there is bias caused by other important factors (Figures 2, 3).

### The Effect Size of Cognitive-Behavioral Training

**FIGURE 4:**
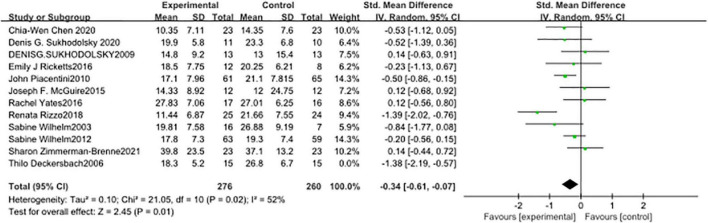
Forest plot of the effect of cognitive-behavioral therapy (CBT) treatment groups vs. control groups on the total tic score.

Twelve studies were measured with a random effect model (Figure 4), and the combined SMD was −0.51 (95% CI: −0.80, −0.22; *p* = 0.0005). The heterogeneity test *I*^2^ was 59%.

**FIGURE 5:**
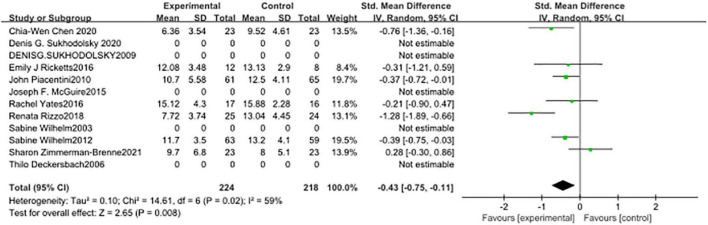
Forest plot of the effect of cognitive-behavioral therapy (CBT) treatment groups vs. control groups on the motor tic score.

**FIGURE 6:**
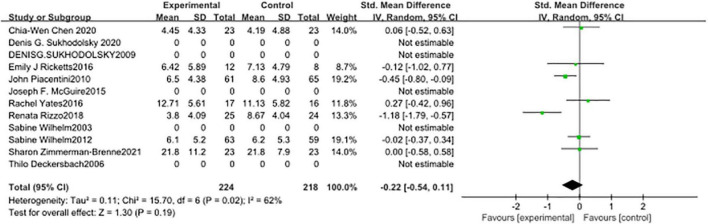
Forest plot of the effect of cognitive-behavioral therapy (CBT) treatment groups vs. control groups on the vocal tic score.

**FIGURE 7:**
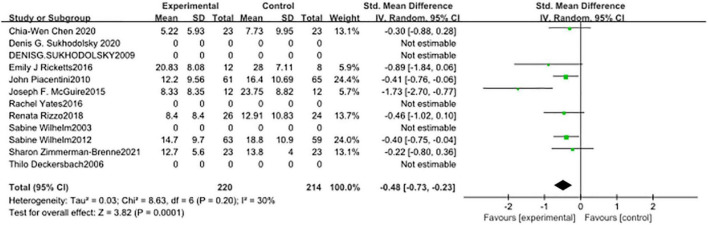
Forest plot of the effect of cognitive-behavioral therapy (CBT) treatment groups vs. control groups on the tic impairment score.

In seven studies (Figure 5), the subjects’ motor tic scores were counted. The sample size of the experimental group was 224 and that of the control group was 218. The pooled SMD was −0.43 (95% CI: −0.75, −0.11; *p* = 0.008), the *I*^2^ was 59%. Seven studies counted the vocal tic scores of subjects (Figure 6), 224 in the experimental group and 218 in the control group. The pooled SMD was −0.22 (95% CI: −0.54, −0.11; *p* = 0.19), the *I*^2^ was 62%. Seven studies counted the tic impairment scores of subjects (Figure 7), 220 in the experimental group and 214 in the control group. The pooled SMD was −0.48 (95% CI: −0.73, −0.23; *p* = 0.0001), the *I*^2^ was 30%.

### Publication Bias

**FIGURE 8:**
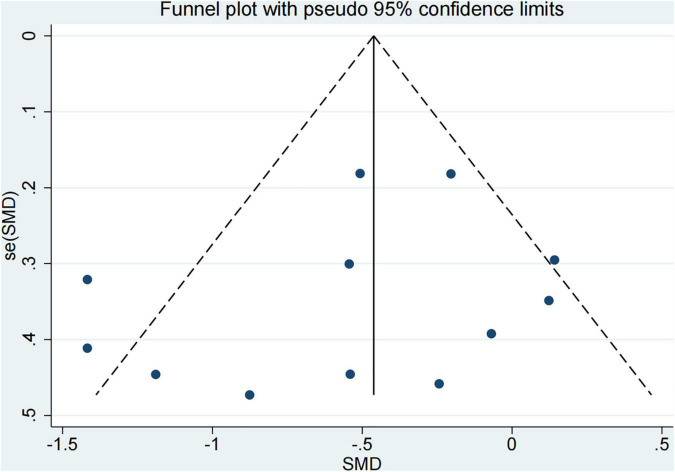
Funnel plot for publication bias evaluation.

The publication bias of these studies was performed by the egger test (Lewis et al., 1997). From Figure 8, there might be no publication bias for included studies.

### Subgroup Analysis

Twelve studies were conducted in six subgroups according to the type of CBT, age, tic severity, country, number of subjects, and publication time. The differences of each group were evaluated by the total score of YGTSS. There was no heterogeneity among the six subgroup analyses (Supplementary Table 1).

## Discussion

At present, the treatment effect of CBT is affirmed in the relevant guidelines of tic disorder in the United States (Murphy et al., 2013), Canada (Steeves et al., 2012), and Europe (Verdellen et al., 2011), and CBT should be the first choice for the treatment of tic disorder (Pringsheim et al., 2019). In this study, the efficacy of CBT was determined through meta-analysis. By summarizing the results of several trials, we believe that CBT is often better than the intervention in the control group to reduce the symptoms of tic disorder. We not only included the latest research, but also used the most detailed subgroup analysis. Subgroup analysis shows that HRT alone seems to reduce patients’ symptoms more than more complex CBIT, and the development of new CBT needs more trials to prove its efficacy. At the same time, according to the current experimental research, there is no significant difference in the therapeutic effect of CBT on patients of different ages, different regions, and different severity. There is no significant difference between studies in different years and different sample sizes.

From the existing research, CBT has become a new direction of tic disorder treatment in the future, and more and more behavioral cognitive therapies are being developed. However, according to the results of this study, there are two problems to be studied in the treatment of tic disorder by CBT. The first problem is whether the more comprehensive the tic disorder CBT is, the better. In this study, the training effect of HRT is better than that of CBIT, which is developed from HRT training. The training method of CBIT includes all the training of HRT, but it is not better than that of HRT in a limited time. There may be three reasons. The first possible reason is that HRT is more accurate for patients, and more time can be spent on training against the emergence of tic movements. Through the existing evaluation methods, the number of tic movements often indicates the severity of tic. In fact, according to the research, tic patients not only suffer from tic disorders but often due to psychological reasons, accompanied by other problems, such as ADHD, obsessive-compulsive disorder (OCD), and so on (March et al., 2007). These can lead to the second reason that why CBIT is not as effective as HRT. The second possible reason may be that HRT can only eliminate the tic movement of patients, and the causes of tic include many other factors, which may be eliminated in CBIT, but the elimination speed is slow, so the training time is not enough to significantly help patients alleviate symptoms. Therefore, more samples were needed to compare HRT with CBIT to verify the efficacy of both. At the same time, more follow-up visits are needed to judge whether the curative effect will continue. In addition, the psychological state of tic disorder patients should be evaluated from multiple dimensions, such as anxiety, fear, and other emotions (Godar and Bortolato, 2016). The second problem of CBT in the treatment of tic disorder is the development of new CBT methods for tic disorder. CBT includes a variety of therapies, far more than HRT and CBIT. At present, the new methods included in this study include ACT, LWT, and neurofeedback. From the research results of this study, the summary analysis of the efficacy of these new methods is not satisfactory. This not only requires more trials to verify its effectiveness but also requires more new methods to be added to the treatment of tic disorder, so as to find more appropriate, more interesting, and more effective treatment methods and reduce the lack of cooperation in the treatment process of tic disorder due to children’s lack of interest, or the effect is not obvious, leads to the non-cooperation of patients and their families.

In the included literature, the bias of Rizzo’s test results is greater than that of other tests (Ricketts et al., 2016). From the data, the effect of this study is stronger than that of other tests. This performance is not only reflected in the evaluation of the total score of tic, but also in the motor score and sound score. The efficacy data of this study are also the best one. According to the intervention measures, it can be found that in the intervention measures, in addition to the regular HRT training courses, each subject is required to carry out HRT training for 15 min under the supervision of their parents every day during HRT training (Ricketts et al., 2016). Taking the training as a part of daily life may be more helpful to form the habit of inhibiting tic disorder movements and sounds. Therefore, the curative effect is higher than in other studies. This method of taking HRT as daily self-training should be paid attention to and applied to more studies to verify its effectiveness for many times. The most effective intervention for tic damage score is Joseph F. McGuire’s LWT intervention method, in which an education module for the subject’s parents is designed to deal with various destructive behaviors made by the subject through corresponding means (McGuire et al., 2015). However, at present, there are few relevant studies on its research methods, which has a certain research prospect, so more research should be carried out on this intervention method. A study has shown that the severity of psychosocial stress can often affect the severity of tic disorder (Lin et al., 2007). In included studies, although some test results are negative, combined with its intervention measures, it may also provide us with some reference significance. For example, in Sharon Zimmerman-Brenne’s study, the CBIT method adopted is different from other studies (Yates et al., 2016). The training is conducted in the form of games in groups of two. It may be that this kind of game aggravates the score of vocal twitch due to the problems reported by the subjects in the process, such as quarrels, which aggravates the large total score of twitch. Chia-Wen Chen and Rachel Yates also showed that HRT and CBIT were not as effective as psychological education groups in the treatment of vocal tic (McGuire et al., 2014; Yu et al., 2020). Therefore, we speculate that if we want to control motor tic, the direction of CBT may need to be biased toward somatic CBT treatment, while the direction of CBT of vocal tic needs to be biased toward psychotherapy. A study in 2014 showed that the factors of TiC have individual differences. The factors of some events can aggravate the tic disorder of one patient, and the same factors can reduce the tic disorder of another patient (Himle et al., 2014). Therefore, the future research direction should not stay in the control of symptoms, but should deeply study their cognitive problems. More targeted investigation, research, and treatment can be carried out in the form of case or case series.

In addition to the above common HRT-based CBT, there is a new CBT method, neurofeedback. Different from other trials, other trials treat by intervening subjects to focus on their own body, while neurofeedback shows subjects the EEG generated by their twitch through visual means. The twitch and twitch control behaviors of patients can be fed back in time, which is convenient for patients to self-supervise at any time. The research shows that this method has achieved certain research results and has certain research potential. More attention should be paid and more experiments should be carried out to verify its effectiveness and prove the authenticity of its research results.

## Limitation

This study has some limitations. Firstly, because there are few studies on some CBT intervention schemes, the conclusion of comparing the effects of various CBTs may not be reliable. Secondly, as a scale-based research, it is often inevitably affected by the evaluator’s supervisor emotion. Thirdly, the emergence of psychological state, family environment, and other factors. Fourth, this study only includes the study of language as English. In the future research, if the research studies of multiple countries and languages can be combined, it may better show the real situation of the disease.

## Conclusion

In short, through this study, it can be seen that the effects of different CBT treatments are slightly different, but in general, it is an effective intervention and a promising treatment. The condition of tic disorder has its unique characteristics. It belongs to a chronic disease with a long course of the disease. Therefore, it is very important whether the treatment effect can be maintained at a certain level. However, the current research is insufficient for the reexamination after treatment. At the same time, there are still insufficient studies on CBT as an intervention method, but from the only remaining studies, It can still be seen that the effectiveness and persistence of CBT intervention are more suitable as the first choice of treatment than drug intervention, and there are still many kinds of CBT intervention methods that have not been used in the treatment of tic disorder, so more trials should be done. At the same time, strengthen the case study of tic disorder.

## Data Availability Statement

The datasets presented in this study can be found in online repositories. The names of the repository/repositories and accession number(s) can be found in the article/Supplementary Material.

## Author Contributions

SS concepted and designed the study. SS and YL contributed to acquisition of data. GF and QZ analyzed the data. SS, YY, and TL drafted and/or revised the manuscript. JW contributed to final approval of the manuscript. All authors contributed to the article and approved the submitted version.

## Conflict of Interest

The authors declare that the research was conducted in the absence of any commercial or financial relationships that could be construed as a potential conflict of interest.

## Publisher’s Note

All claims expressed in this article are solely those of the authors and do not necessarily represent those of their affiliated organizations, or those of the publisher, the editors and the reviewers. Any product that may be evaluated in this article, or claim that may be made by its manufacturer, is not guaranteed or endorsed by the publisher.

## References

[B1] American Psychiatric Association [APA] (2013). *The Diagnostic and Statistical Manual of Mental Disorders (DSM-5)*, 5th Edn. Virginia Beach, VA: American Psychiatric Association.

[B2] Azrin N. H. Nunn R. G. (1973). Habit-reversal: a method of eliminating nervous habits and tics. *Behav. Res. Ther.* 11 619–628. 10.1016/0005-7967(73)90119-8 4777653

[B3] Bate K. S. Malouff J. M. Thorsteinsson E. T. Bhullar N. (2011). The efficacy of habit reversal therapy for tics, habit disorders, and stuttering: a meta-analytic review. *Clin. Psychol. Rev.* 31 865–871. 10.1016/j.cpr.2011.03.013 21549664

[B4] Bressan R. A. Jones H. M. Pilowsky L. S. (2004). Atypical antipsychotic drugs and tardive dyskinesia: relevance of D2 receptor affinity. *J. Psychopharmacol.* 18 124–127. 10.1177/0269881104040251 15107196

[B5] Chadehumbe M. A. Greydanus D. E. Feucht C. Patel D. R. (2011). Psychopharmacology of tic disorders in children and adolescents. *Pediatr. Clin. North Am.* 58 259–272. 10.1016/j.pcl.2010.10.004 21281860

[B6] Chen C. W. Wang H. S. Chang H. J. Hsueh C. W. (2020). Effectiveness of a modified comprehensive behavioral intervention for tics for children and adolescents with tourette’s syndrome: a randomized controlled trial. *J. Adv. Nurs.* 76 903–915. 10.1111/jan.14279 31782167

[B7] Curtis A. Clarke C. E. Rickards H. E. (2009). Cannabinoids for Tourette’s syndrome. *Cochrane Database Syst. Rev.* 2009:CD006565. 10.1002/14651858.CD006565.pub2 19821373 PMC7387115

[B8] Davis K. K. Davis J. S. Dowler L. (2004). In motion, out of place: the public space(s) of Tourette Syndrome. *Soc. Sci. Med.* 59 103–112. 10.1016/j.socscimed.2003.10.008 15087147

[B9] Deckersbach T. Rauch S. Buhlmann U. Wilhelm S. (2006). Habit reversal versus supportive psychotherapy in Tourette’s disorder: a randomized controlled trial and predictors of treatment response. *Behav. Res. Ther.* 44 1079–1090. 10.1016/j.brat.2005.08.007 16259942

[B10] Gaffney G. R. Perry P. J. Lund B. C. Bever-Stille K. A. Arndt S. Kuperman S. (2002). Risperidone versus clonidine in the treatment of children and adolescents with Tourette’s syndrome. *J. Am. Acad. Child. Adolesc. Psychiatry* 41 330–336. 10.1097/00004583-200203000-00013 11886028

[B11] Ganos C. Neumann W. J. Müller-Vahl K. R. Bhatia K. P. Hallett M. Haggard P. (2021). The phenomenon of exquisite motor control in tic disorders and its pathophysiological implications. *Mov. Disord.* 36 1308–1315. 10.1002/mds.28557 33739492

[B12] Godar S. C. Bortolato M. (2016). What makes you tic? Translational approaches to study the role of stress and contextual triggers in Tourette syndrome. *Neurosci. Biobehav. Rev.* 76 123–133. 10.1016/j.neubiorev.2016.10.003 27939782 PMC5403589

[B13] Goetz C. G. (1992). Clonidine and clonazepam in Tourette syndrome. *Adv. Neurol.* 58 245–251. 1414629

[B14] Higgins J. Thomopson S. G. Deeks J. J. Altman D. G. (2008). *Cochrane Handbook for Systematic Reviews of Interventions version 5.1.0.* Chichester: John Wiley & Sons.

[B15] Himle M. B. Capriotti M. R. Hayes L. P. Ramanujam K. Scahill L. Sukhodolsky D. G. (2014). Variables associated with tic exacerbation in children with chronic tic disorders. *Behav. Modif.* 38 163–183. 10.1177/0145445514531016 24778433 PMC4211980

[B16] Lewis S. J. Zammit S. Gunnell D. Smith G. D. (1997). *Bias in Meta-Analysis Detected by a Simple, Graphical Test, Wiley Subscription Services, Inc.* Hoboken, NJ: Wiley.

[B17] Lin H. Katsovich L. Ghebremichael M. Findley D. B. Grantz H. Lombroso P. J. (2007). Psychosocial stress predicts future symptom severities in children and adolescents with Tourette syndrome and/or obsessive-compulsive disorder. *J. Child. Psychol. Psychiatry* 48 157–166. 10.1111/j.1469-7610.2006.01687.x 17300554 PMC3073143

[B18] March J. S. Franklin M. E. Leonard H. Garcia A. Moore P. Freeman J. (2007). Tics moderate treatment outcome with sertraline but not cognitive-behavior therapy in pediatric obsessive-compulsive disorder. *Biol. Psychiatry* 61 344–347. 10.1016/j.biopsych.2006.09.035 17241830

[B19] McGuire J. F. Arnold E. Park J. M. Nadeau J. M. Lewin A. B. Murphy T. K. (2015). Living with tics: reduced impairment and improved quality of life for youth with chronic tic disorders. *Psychiatry Res.* 225 571–579. 10.1016/j.psychres.2014.11.045 25500348 PMC4314444

[B20] McGuire J. F. Piacentini J. Brennan E. A. Lewin A. B. Murphy T. K. Small B. J. (2014). A meta-analysis of behavior therapy for Tourette Syndrome. *J. Psychiatr. Res.* 50 106–112. 10.1016/j.jpsychires.2013.12.009 24398255

[B21] McNaught K. S. Mink J. W. (2011). Advances in understanding and treatment of Tourette syndrome. *Nat. Rev. Neurol.* 7 667–676. 22064610 10.1038/nrneurol.2011.167

[B22] Murphy T. K. Lewin A. B. Storch E. A. Stock S. L. (2013). Practice parameter for the assessment and treatment of children and adolescents with tic disorders. *J. Am. Acad. Child. Adolesc. Psychiatry* 52 1341–1359. 10.1016/j.jaac.2013.09.015 24290467

[B23] O’Donohue W. T. Fisher J. E. Hayes S. C. (2003). *Cognitive Behavior Therapy: Applying Empirically Supported Techniques in Your Practice*, 2nd Edn. Hoboken, NJ: Wiley.

[B24] O’Hare D. Helmes E. Eapen V. Grove R. McBain K. Reece J. (2016). The impact of tic severity, comorbidity and peer attachment on quality of life outcomes and functioning in Tourette’s syndrome: parental perspectives. *Child. Psychiatry Hum. Dev.* 47 563–573. 10.1007/s10578-015-0590-7 26440978

[B25] Piacentini J. Woods D. W. Scahill L. Wilhelm S. Peterson A. L. Chang S. (2010). Behavior therapy for children with Tourette disorder: a randomized controlled trial. *JAMA* 303 1929–1937. 10.1001/jama.2010.607 20483969 PMC2993317

[B26] Pringsheim T. Okun M. S. Müller-Vahl K. Martino D. Jankovic J. Cavanna A. E. (2019). Practice guideline recommendations summary: treatment of tics in people with Tourette syndrome and chronic tic disorders. *Neurology* 92 896–906. 10.1212/WNL.0000000000007466 31061208 PMC6537133

[B27] Ricketts E. J. Goetz A. R. Capriotti M. R. Bauer C. C. Brei N. G. Himle M. B. (2016). A randomized waitlist-controlled pilot trial of voice over Internet protocol-delivered behavior therapy for youth with chronic tic disorders. *J. Telemed. Telecare* 22 153–162. 10.1177/1357633X15593192 26169350 PMC6033263

[B28] Rizzo R. Pellico A. Silvestri P. R. Chiarotti F. Cardona F. (2018). A Randomized Controlled Trial Comparing Behavioral, Educational, and Pharmacological Treatments in Youths With Chronic Tic Disorder or Tourette Syndrome. *Front. Psychiatry* 9:100. 10.3389/fpsyt.2018.00100 29636706 PMC5880916

[B29] Roessner V. Plessen K. J. Rothenberger A. Ludolph A. G. RizzoI R. Skov L. (2011). European clinical guidelines for Tourette syndrome and other tic disorders. Part II: pharmacological treatment. *Eur. Child Adolesc. Psychiatry* 20 173–196. 10.1007/s00787-011-0163-7 21445724 PMC3065650

[B30] Romero K. Pavisian B. Staines W. R. Feinstein A. (2015). Multiple sclerosis, cannabis, and cognition: a structural MRI study. *Neuroimage Clin.* 8 140–147. 10.1016/j.nicl.2015.04.006 26106538 PMC4473732

[B31] Song P. P. Jiang L. Li X. J. HongG S. Q. Li S. Z. Hu Y. (2017). The efficacy and tolerability of the clonidine transdermal patch in the treatment for children with tic disorders: a prospective, open, single-group, self-controlled study. *Front. Neurol.* 8:32. 10.3389/fneur.2017.00032 28280480 PMC5322220

[B32] Steeves T. McKinlay B. D. Gorman D. Billinghurst L. Day L. Carroll A. (2012). Canadian guidelines for the evidence-based treatment of tic disorders: behavioural therapy, deep brain stimulation, and transcranial magnetic stimulation. *Can. J. Psychiatry* 57 144–151. 22398000 10.1177/070674371205700303

[B33] Sukhodolsky D. G. Vitulano L. A. Carroll D. H. McGuire J. Leckman J. F. Scahill L. (2009). Randomized trial of anger control training for adolescents with Tourette’s syndrome and disruptive behavior. *J. Am. Acad. Child. Adolesc. Psychiatry* 48 413–421. 10.1097/CHI.0b013e3181985050 19242384 PMC13127727

[B34] Sukhodolsky D. G. Walsh C. Koller W. N. EilbNott J. Rance M. Fulbright R. K. (2020). Randomized, sham-controlled trial of real-time functional magnetic resonance imaging neurofeedback for tics in adolescents with Tourette syndrome. *Biol. Psychiatry* 87 1063–1070. 10.1016/j.biopsych.2019.07.035 31668476 PMC7015800

[B35] Teng E. J. Woods D. W. Twohig M. P. (2006). Habit reversal as a treatment for chronic skin picking: a pilot investigation. *Behav. Modif.* 30 411–422. 10.1177/0145445504265707 16723422

[B36] Verdellen C. van de Griendt J. Hartmann A. Murphy T. Essts Guidelines Group. (2011). European clinical guidelines for Tourette syndrome and other tic disorders. Part III: behavioural and psychosocial interventions. *Eur. Child. Adolesc. Psychiatry* 20 197–207. 10.1007/s00787-011-0167-3 21445725

[B37] Weisman H. Qureshi I. A. Leckman J. F. Scahill L. Bloch M. H. (2013). Systematic review: pharmacological treatment of tic disorders–efficacy of antipsychotic and alpha-2 adrenergic agonist agents. *Neurosci. Biobehav. Rev.* 37 1162–1171. 10.1016/j.neubiorev.2012.09.008 23099282 PMC3674207

[B38] Wilhelm S. Deckersbach T. Coffey B. J. Bohne A. Peterson A. L. Baer L. (2003). Habit reversal versus supportive psychotherapy for Tourette’s disorder: a randomized controlled trial. *Am. J. Psychiatry* 160 1175–1177. 12777279 10.1176/appi.ajp.160.6.1175

[B39] Wilhelm S. Peterson A. L. Piacentini J. Woods D. W. Deckersbach T. Sukhodolsky D. G. (2012). Randomized trial of behavior therapy for adults with Tourette syndrome. *Arch. Gen. Psychiatry* 69 795–803. 10.1001/archgenpsychiatry.2011.1528 22868933 PMC3772729

[B40] Yates R. Edwards K. King J. Luzon O. Evangeli M. Stark D. (2016). Habit Reversal Training and Educational group treatments for children with Tourette syndrome: a preliminary randomised controlled trial. *Behav. Res. Ther.* 80 43–50. 10.1016/j.brat.2016.03.003 27037483

[B41] Yu L. Li Y. Zhang J. Yan C. Wen F. Yan J. (2020). The therapeutic effect of habit reversal training frome: a meta-analysis of randomized control trials. *Expert Rev. Neurother.* 20 1189–1196. 10.1080/14737175.2020.1826933 32948114

[B42] Zimmerman-Brenner S. Pilowsky-Peleg T. Rachamim L. Ben-Zvi A. Gur N. Murphy T. (2021). Group behavioral interventions for tics and comorbid symptoms in children with chronic tic disorders. *Eur. Child. Adolesc. Psychiatry.* [Epub ahead of print]. 10.1007/s00787-020-01702-5 33415472

