# Supplementary material for: The Efficacy of Cognitive Behavioral Therapy for Tic Disorder: A Meta-Analysis and a Literature Review
Source: Front Psychol. 2022 Mar 24;13:851250. doi: 10.3389/fpsyg.2022.851250 (PMC8987272; doi:10.3389/fpsyg.2022.851250)
Supplement: Supplementary file 1 [file Table_1.doc]

**Medline search strategy：**((((((((((((((((((((((((((((((((((((((((((((((((((((((((((((((((((((((((((((((((((((((((((((((((((((((((((((((((((((((((((((((((((((((((((((((((((((((((((((((((((((((((((((((((((((((((((((((((((((((((((Behavior Modification[Title/Abstract]) OR (Therapy, Conditioning[Title/Abstract])) OR (Behavior Therapies[Title/Abstract])) OR (Therapy, Conditioning[Title/Abstract])) OR (Conditioning Therapy[Title/Abstract])) OR (Conditioning Therapies[Title/Abstract])) OR (Therapy, Behavior[Title/Abstract])) OR (Behavior Treatment[Title/Abstract])) OR (Treatment, Behavior[Title/Abstract])) OR (Behavior Modification[Title/Abstract])) OR (Behavior Modifications[Title/Abstract])) OR (Modification, Behavior[Title/Abstract])) OR (Therapy, Anger Management[Title/Abstract])) OR (Anger Management Therapy[Title/Abstract])) OR (Anger Management Training[Title/Abstract])) OR (Management Training, Anger[Title/Abstract])) OR (Training, Anger Management[Title/Abstract])) OR (Management, Anger[Title/Abstract])) OR (Anger Management[Title/Abstract])) OR (Analyses, Applied Behavior[Title/Abstract])) OR (Applied Behavior Analyses[Title/Abstract])) OR (Behavior Analyses, Applied[Title/Abstract])) OR (Behavior Analysis, Applied[Title/Abstract])) OR (Applied Behavior Analysis[Title/Abstract])) OR (Aversive Therapy[Title/Abstract])) OR (Analyses, Applied Behavior[Title/Abstract])) ) OR (Biofeedbacks, Psychology[Title/Abstract])) OR (Psychology Biofeedback[Title/Abstract])) OR (Psychology Biofeedbacks[Title/Abstract])) OR (Psychophysiologic Feedback[Title/Abstract])) OR (Feedback, Psychophysiologic[Title/Abstract])) OR (Feedback, Psychophysiological[Title/Abstract])) OR (Biofeedback[Title/Abstract])) OR (Biofeedbacks[Title/Abstract])) OR (Myofeedback[Title/Abstract])) OR (Myofeedbacks[Title/Abstract])) OR (False Physiological Feedback[Title/Abstract])) OR (False Physiological Feedbacks[Title/Abstract])) OR (Feedback, False Physiological[Title/Abstract])) OR (Feedbacks, False Physiological[Title/Abstract])) OR (Physiological Feedback, False[Title/Abstract])) OR (Physiological Feedbacks, False[Title/Abstract])) OR (Bogus Physiological Feedback[Title/Abstract])) OR (Bogus Physiological Feedbacks[Title/Abstract])) OR (Feedback, Bogus Physiological[Title/Abstract])) OR (Feedbacks, Bogus Physiological[Title/Abstract])) OR (Physiological Feedback, Bogus[Title/Abstract])) OR (Physiological Feedbacks, Bogus[Title/Abstract])) OR (Feedback, Sensory[Title/Abstract])) OR (Feedbacks, Sensory[Title/Abstract])) OR (Sensory Feedback[Title/Abstract])) OR (Sensory Feedbacks[Title/Abstract])) OR (Visual Feedback[Title/Abstract])) OR (Feedback, Visual[Title/Abstract])) OR (Feedbacks, Visual[Title/Abstract])) OR (Visual Feedbacks[Title/Abstract])) OR (Sensorimotor Feedback[Title/Abstract])) OR (Feedback, Sensorimotor[Title/Abstract])) OR (Feedbacks, Sensorimotor[Title/Abstract])) OR (Sensorimotor Feedbacks[Title/Abstract])) OR (Audio Feedback[Title/Abstract])) OR (Audio Feedbacks[Title/Abstract])) OR (Feedback, Audio[Title/Abstract])) OR (Feedbacks, Audio[Title/Abstract])) OR (Proprioceptive Feedback[Title/Abstract])) OR (Feedback, Proprioceptive[Title/Abstract])) OR (Feedbacks, Proprioceptive[Title/Abstract])) OR (Proprioceptive Feedbacks[Title/Abstract])) OR (Neurofeedback[Title/Abstract])) OR (Neurofeedbacks[Title/Abstract])) OR (Brainwave Biofeedback[Title/Abstract])) OR (Biofeedback, Brainwave[Title/Abstract])) OR (Biofeedbacks, Brainwave[Title/Abstract])) OR (Brainwave Biofeedbacks[Title/Abstract])) OR (Alpha Feedback[Title/Abstract])) OR (Alpha Feedbacks[Title/Abstract])) OR (Feedback, Alpha[Title/Abstract])) OR (Feedbacks, Alpha[Title/Abstract])) OR (Electromyography Feedback[Title/Abstract])) OR (EEG Feedback[Title/Abstract])) OR (EEG Feedbacks[Title/Abstract])) OR (Feedback, EEG[Title/Abstract])) OR (Feedbacks, EEG[Title/Abstract])) OR (Electroencephalography Biofeedback[Title/Abstract])) OR (Biofeedback, Electroencephalography[Title/Abstract])) OR (Biofeedbacks, Electroencephalography[Title/Abstract])) OR (Electroencephalography Biofeedbacks[Title/Abstract])) OR (Alpha Biofeedback[Title/Abstract])) OR (Alpha Biofeedbacks[Title/Abstract])) OR (Biofeedback, Alpha[Title/Abstract])) OR (Biofeedbacks, Alpha[Title/Abstract])) OR (Brainwave Feedback[Title/Abstract])) OR (Brainwave Feedbacks[Title/Abstract])) OR (Feedback, Brainwave[Title/Abstract])) OR (Feedbacks, Brainwave[Title/Abstract])) OR (Cognitive Behavioral Therapy[Title/Abstract])) OR (Behavioral Therapies, Cognitive[Title/Abstract])) OR (Behavioral Therapy, Cognitive[Title/Abstract])) OR (Cognitive Behavioral Therapies[Title/Abstract])) OR (Therapies, Cognitive Behavioral[Title/Abstract])) OR (Therapy, Cognitive Behavioral[Title/Abstract])) OR (Therapy, Cognitive Behavior[Title/Abstract])) OR (Cognitive Behavior Therapy[Title/Abstract])) OR (Cognitive Therapy[Title/Abstract])) OR (Behavior Therapy, Cognitive[Title/Abstract])) OR (Behavior Therapies, Cognitive[Title/Abstract])) OR (Cognitive Behavior Therapies[Title/Abstract])) OR (Therapies, Cognitive Behavior[Title/Abstract])) OR (Cognitive Psychotherapy[Title/Abstract])) OR (Cognitive Psychotherapies[Title/Abstract])) OR (Psychotherapies, Cognitive[Title/Abstract])) OR (Psychotherapy, Cognitive[Title/Abstract])) OR (Therapy, Cognitive[Title/Abstract])) OR (Cognitive Therapies[Title/Abstract])) OR (Therapies, Cognitive[Title/Abstract])) OR (Cognition Therapy[Title/Abstract])) OR (Therapy, Cognition[Title/Abstract])) OR (Cognition Therapies[Title/Abstract])) OR (Therapies, Cognition[Title/Abstract])) OR (Acceptance[Title/Abstract] AND Commitment Therapy[Title/Abstract])) OR (Mindfulness[Title/Abstract])) OR (Cognitive Remediation[Title/Abstract])) OR (Remediation, Cognitive[Title/Abstract])) OR (Desensitization, Psychologic[Title/Abstract])) OR (Psychologic Desensitization[Title/Abstract])) OR (Psychological Desensitization[Title/Abstract])) OR (Desensitization, Psychological[Title/Abstract])) OR (Desensitizations [Title/Abstract])) OR (Desensitization [Title/Abstract])) OR (Eye Movement Desensitization Reprocessing[Title/Abstract])) OR (EMDR[Title/Abstract])) OR (Eye Movement Desensitization[Title/Abstract] AND Processing[Title/Abstract])) OR (Implosive Therapy[Title/Abstract])) OR (Implosive Therapies[Title/Abstract])) OR (Therapies, Implosive[Title/Abstract])) OR (Flooding, Imaginal[Title/Abstract])) OR (Floodings, Imaginal[Title/Abstract])) OR (Imaginal Floodings[Title/Abstract])) OR (Therapy, Implosive[Title/Abstract])) OR (Flooding Therapies[Title/Abstract])) OR (Flooding Therapy[Title/Abstract])) OR (Therapies, Flooding[Title/Abstract])) OR (Therapy, Flooding[Title/Abstract])) OR (Flooding Therapies[Title/Abstract])) OR (Therapies, Flooding[Title/Abstract])) OR (Therapy, Flooding[Title/Abstract])) OR (Imaginal Flooding[Title/Abstract])) OR (Exposure Therapy[Title/Abstract])) OR (Exposure Therapies[Title/Abstract])) OR (Therapies, Exposure[Title/Abstract])) OR (Therapy, Exposure[Title/Abstract])) OR (Virtual Reality Exposure Therapy[Title/Abstract])) OR (Virtual Reality Immersion Therapy[Title/Abstract])) OR (Virtual Reality Therapy[Title/Abstract])) OR (Reality Therapies, Virtual[Title/Abstract])) OR (Reality Therapy, Virtual[Title/Abstract])) OR (Therapies, Virtual Reality[Title/Abstract])) OR (Therapy, Virtual Reality[Title/Abstract])) OR (Virtual Reality Therapies[Title/Abstract])) OR (Dialectical Behavior Therapy[Title/Abstract])) OR (Behavior Therapy, Dialectical[Title/Abstract])) OR (Dialectical Behavior Therapies[Title/Abstract])) OR (Relaxation Therapy[Title/Abstract])) OR (Therapy, Relaxation[Title/Abstract])) OR (Therapeutic Relaxation[Title/Abstract])) OR (Relaxation, Therapeutic[Title/Abstract])) OR (Relaxation Techniques[Title/Abstract])) OR (Relaxation Technique[Title/Abstract])) OR (Technique, Relaxation[Title/Abstract])) OR (Techniques, Relaxation[Title/Abstract])) OR (Relaxation Technics[Title/Abstract])) OR (Relaxation Technic[Title/Abstract])) OR (Technic, Relaxation[Title/Abstract])) OR (Nature Therapy[Title/Abstract])) OR (Nature Therapies[Title/Abstract])) OR (Therapy, Nature[Title/Abstract])) OR (Ecotherapy[Title/Abstract])) OR (Ecotherapies[Title/Abstract])) OR (Meditation[Title/Abstract])) OR (Transcendental Meditation[Title/Abstract])) OR (Meditation, Transcendental[Title/Abstract])) OR (Sleep Phase Chronotherapy[Title/Abstract])) OR (Chronotherapies, Sleep Phase[Title/Abstract])) OR (Chronotherapy, Sleep Phase[Title/Abstract])) OR (Sleep Phase Chronotherapies[Title/Abstract])) OR (Phase Advance Chronotherapy[Title/Abstract])) OR (Advance Chronotherapies, Phase[Title/Abstract])) OR (Advance Chronotherapy, Phase[Title/Abstract])) OR (Chronotherapies, Phase Advance[Title/Abstract])) OR (Chronotherapy, Phase Advance[Title/Abstract])) OR (Phase Advance Chronotherapies[Title/Abstract])) OR (Phase Delay Chronotherapy[Title/Abstract])) OR (Chronotherapies, Phase Delay[Title/Abstract])) OR (Chronotherapy, Phase Delay[Title/Abstract])) OR (Delay Chronotherapies, Phase[Title/Abstract])) OR (Delay Chronotherapy, Phase[Title/Abstract])) OR (Phase Delay Chronotherapies[Title/Abstract])

Supplement Table 1:Analysis of 6 subgroups

| Basis of grouping | Subgroup name | SMD | Heterogeneity of each group | Intergroup heterogeneity |
| --- | --- | --- | --- | --- |
| CBT type | HRT  CBIT  Other CBT | -0.87[-1.63,-0.11]  -0.30[-0.53,-0.08]  -0.55[-0.53,0.07] | P=0.02, I^2^=76%  P=0.008, I^2^=9%  P=0.08, I^2^=40% | I^2^=14.7% |
| Age | Juveniles  Adult | -0.45 [-0.79, -0.12]  -0.74[-1.51,-0.03] | P=0.008, I^2^=58%  P=0.06, I^2^=74% | I^2^=0% |
| Tic severity | Tic score<25  Tic score>25 | -0.74 [-1.51, 0.03]  -0.74 [-1.51, 0.03] | P=0.002, I^2^=56%  P=0.15, I^2^=75% | I^2^=0% |
| Country | USA  Other country | -0.53 [-0.82, -0.24]  -0.42 [-1.12, 0.28] | P=0.0004, I^2^=38%  P=0.24, I_2_=80% | I^2^=0% |
| Number of subjects | >20  <20 | -0.47 [-0.88, -0.06]  -0.56 [-1.00, -0.12] | P=0.02, I^2^=73%  P=0.01, I^2^=51% | I^2^=0% |
| Publication time | 2000-2009  2010-2019  2020-2021 | -0.75 [-1.54, 0.04]  -0.53 [-0.95, -0.12]  -0.27[0.73,-0.20] | P=0.06, I^2^=63%  P=0.01, I^2^=69%  P=0.26, I^2^=33% | I^2^=0% |
